# Canadian Veteran chronic disease prevalence and health services use in the five years following release: a matched retrospective cohort study using routinely collected data

**DOI:** 10.1186/s12889-022-14053-4

**Published:** 2022-09-05

**Authors:** Alyson L. Mahar, Kate St. Cyr, Jennifer E. Enns, Alice B. Aiken, Marlo Whitehead, Heidi Cramm, Paul Kurdyak

**Affiliations:** 1 ICES, Toronto, Canada; 2 Manitoba Centre for Health Policy, Department of Community Health Sciences, University of Manitoba, Winnipeg, Canada; 3 Dalla Lana School of Public Health, University of Toronto, Toronto, Canada; 4 Faculty of Health, Dalhousie University, Halifax, Canada; 5 School of Rehabilitation Therapy, Queen’s University, Kingston, Canada; 6 Institute for Health Policy, Management, and Evaluation, University of Toronto, Toronto, Canada

**Keywords:** Canadian Armed Forces, Veterans, Epidemiology, Chronic disease, Health services, Population health, Cohort study

## Abstract

**Background:**

Occupational exposures may result in Canadian military Veterans having poorer health and higher use of health services after transitioning to civilian life compared to the general population. However, few studies have documented the physical health and health services use of Veterans in Canada, and thus there is limited evidence to inform public health policy and resource allocation.

**Methods:**

In a retrospective, matched cohort of Veterans and the Ontario general population between 1990–2019, we used routinely collected provincial administrative health data to examine chronic disease prevalence and health service use. Veterans were defined as former members of the Canadian Armed Forces or RCMP. Crude and adjusted effect estimates, and 95% confidence limits were calculated using logistic regression (asthma, COPD, diabetes, myocardial infarction, rheumatoid arthritis, family physician, specialist, emergency department, and home care visits, as well as hospitalizations). Modified Poisson was used to estimate relative differences in the prevalence of hypertension. Poisson regression compares rates of health services use between the two groups.

**Results:**

The study included 30,576 Veterans and 122,293 matched civilians. In the first five years after transition to civilian life, Veterans were less likely than the general population to experience asthma (RR 0.50, 95% CI 0.48–0.53), COPD (RR 0.32, 95% CI 0.29–0.36), hypertension (RR 0.74, 95% CI 0.71–0.76), diabetes (RR 0.71, 95% CI 0.67–0.76), myocardial infarction (RR 0.76, 95% CI 0.63–0.92), and rheumatoid arthritis (RR 0.74, 95% CI 0.60–0.92). Compared to the general population, Veterans had greater odds of visiting a primary care physician (OR 1.76, 95% CI 1.70–1.83) or specialist physician (OR 1.39, 95% CI 1.35–1.42) at least once in the five-year period and lower odds of visiting the emergency department (OR 0.95, 95% CI 0.92–0.97). Risks of hospitalization and of receiving home care services were similar in both groups.

**Conclusions:**

Despite a lower burden of comorbidities, Veterans had slightly higher physician visit rates. While these visits may reflect an underlying need for services, our findings suggest that Canadian Veterans have good access to primary and specialty health care. But in light of contradictory findings in other jurisdictions, the underlying reasons for our findings warrant further study.

**Supplementary Information:**

The online version contains supplementary material available at 10.1186/s12889-022-14053-4.

## Background

Globally, there are approximately 17 million military Veterans living in the United States [1], over 2 million armed forces Veterans residing in the United Kingdom [2], and approximately 597,200 Canadian Armed Forces (CAF) Veterans living in Canada [3]. During their military service, Veterans are exposed to a variety of unique occupational hazards (e.g., deployment to war zones, physically demanding tasks, etc.), placing them at risk for service-related injury, illness, and disability (e.g., traumatic brain injury, mental illness, limb amputation, etc.) [4, 5]. Thus, a substantial proportion of Veterans are also clients of Veterans Affairs Canada (VAC) [3], meaning that they receive additional support or benefits from VAC. While employed in the military, CAF members receive customized healthcare through the Department of National Defence to maintain a level of health and wellness that meets employer standards. However, the approximately 4,000–5,000 CAF members (and a comparative number of reserve force members) who are released each year receive the majority of their healthcare from civilian healthcare professionals in provincial and territorial health systems [6]. This differs from the US, which provides health services to all eligible Veterans in hospitals, clinics, counseling centres and long-term care facilities separate from the private healthcare system and funded by the Department of Veterans Affairs [7].

While there is a wealth of data from the US [8–10] and other countries [11, 12] supporting the delivery of evidence-based healthcare to Veterans, information on the health and health services use patterns of Canadian Veterans is limited. Research from the US demonstrates that Veterans have higher rates of diabetes [13, 14], chronic obstructive pulmonary disease (COPD) [14, 15], arthritis [16, 17], high blood pressure [14, 18], and cancer [14, 17] than members of the general population. In the UK and Australia, Veterans have also been reported to have higher rates of cardiovascular disease [19–21]. However, military operations, military lifestyle, and military and post-service healthcare systems can vary significantly between countries and the underlying prevalence of chronic disease and health services use may also vary between countries. As such, Canada-specific data are needed to accurately inform healthcare policy and resource allocation for Veterans.

Only a small number of studies on post-discharge health risks and healthcare seeking behaviours among Canadian Veterans exist. Most of these studies focus on mental health [22–24], and the remainder rarely compare risks directly with the general population or measure disease or health service use status using administrative health system data. For example, a 2016 Veterans Affairs Canada and Statistics Canada-administered survey found that, compared to the 2013/14 Canadian general population, CAF Veterans reported higher prevalence of arthritis and cancer but similar or lower prevalence of chronic obstructive pulmonary disease (COPD), asthma, and diabetes [25]. Although a similar proportion of Veterans reported having a regular medical doctor and consulting with a family doctor or a specialist in the previous year as the general population, these findings are thought to be an underestimate of need given the higher rates of disability, chronic pain and other chronic illness among Veterans [26–28]. However, this survey relied on self-reported data from a small national sample of Veterans (*n* = 2,755) and may not be representative of Canadian Veterans residing in Ontario. Population-based routinely collected administrative health data have considerable advantages over survey data for estimating the prevalence of physical health conditions and the rates of health service use amongst Canadian Veterans relative to the general population. Primary amongst these is that studies using de-identified population-based administrative data do not rely on a sample, but instead capture the whole population of interest. Administrative data also do not rely on self-report, avoiding participation bias and recall bias in survey responses.

## Methods

### Study aim, design and setting

In this study, we used population-based administrative health and health services data from Ontario to compare the prevalence of health conditions (asthma, COPD, diabetes, hypertension, myocardial infarction, and rheumatoid arthritis) and health services use, defined as use of primary care, specialist care, emergency department visits, hospitalizations, and home care by CAF Veterans with the general population of Ontario, Canada. This information will be valuable to Canadian healthcare planners and providers in managing the health of Veterans in their home communities.

We used a retrospective, matched cohort design of Veterans and the Ontario general population. Ontario is the most populous province in Canada, with an estimated 14.7 million inhabitants as of March 31, 2020 [29]. It is also home to eight CAF bases, the Royal Military College of Canada, the Department of National Defence Headquarters, and the Royal Canadian Mounted Police (RCMP) headquarters [23]. This study received ethics approval from the University of Manitoba’s Health Research Ethics Board (protocol number HS22485).

### Data sources

The data for this study are held at ICES (formerly the Institute for Clinical Evaluative Sciences), a not-for-profit health services and policy research institute that provides stewardship over Ontario’s administrative health data. We linked the following ICES administrative datasets at the individual level using unique encoded identifiers: the Ontario Health Insurance Plan (OHIP) database (enrolment in provincial health insurance plan, physician billing records); the Ontario Drug Benefit database (enrolment in income support programs, long-term care stay); the National Rehabilitation Reporting System (rehabilitation stay); the Registered Persons Database (sociodemographic data, including Veteran status, age, sex, residential geography, neighbourhood median income, date of death, and end date of OHIP eligibility); the ICES Physician Database (physician specialty); the Canadian Institute of Health Information (CIHI) Discharge Abstract Database and the CIHI-Same Day Surgery databases (hospitalizations, including diagnoses and interventions); the National Ambulatory Care Reporting System (emergency department visits, including diagnostic and service information); and the Home Care database (publicly funded home care services, including those provided by nurses and allied health professionals, and general homemaking services).

### Veteran status

We defined Veterans as former members of the CAF or RCMP who provided evidence of their military service to the Ministry of Health and Long-Term Care (MOHLTC) at the time of enrolment in OHIP. Health insurance coverage transitions from federal to provincial oversight at the time of departure from the CAF and RCMP. In Ontario, standard waiting periods for provincial health insurance are waived when evidence of CAF or RCMP service is provided; an administrative military service code and service start, and end dates are linked to the individual’s provincial health card. The MOHLTC provided an anonymized list of individuals with an administrative military service code linked to their health card number to ICES. Data anonymization, linkage to the unique encoded identifier (ICES Key Number), and removal of the health card number were performed according to standard ICES protocol by the ICES Data Acquisition team. Identifying information was removed from the cohort prior to access by the study authors.

Veterans were included in the study if they registered for OHIP between January 1, 1990, and December 31, 2019. The date of OHIP registration is a close approximation of the Veteran’s release date from the CAF or RCMP [30]. We excluded Veterans who had OHIP coverage while still engaged in CAF or RCMP service, as indicated by OHIP billing record dates, or who were younger than 16 years of age at the start date of military or RCMP service. We have previously compared the representativeness and expected prevalence of Veterans in this cohort to federal and provincial statistics for Veterans and RCMP [30].

### Matched civilian comparator cohorts

Veterans were matched with up to four members of the general population with replacement. Each Veteran’s OHIP registration date was used as the index date for the matched civilian reference groups. Eligible members of the general population were alive at the study index date. To reduce the likelihood of the healthy worker effect, where people who are employed generally experience lower mortality and morbidity than the general population (which includes those who cannot work due to disability or illness) [31], we selected members of the general population most likely to be employed during the period of military or RCMP service of the matched Veteran. As a result, we excluded members of the general population who had a long-term care stay, attended a rehabilitation facility, or received disability or income support during the period in which they would have been eligible for military service. The general population cohort was hard matched on age (birth year), sex, residential geography, and median neighbourhood income quintile in the index year. Individuals were assigned to one of fourteen geographic regions previously used for healthcare planning and provision based on their postal code. Median neighbourhood income quintile was derived from postal code and Canada Census information.

### Outcome variables

The study had two primary outcome categories: chronic disease prevalence and health service use. Both categories were measured in the five-year period following the index date. Persons were followed until end of OHIP coverage (e.g., moved out of province), death, or until the end of the study period (December 31, 2019). Asthma, COPD, diabetes, hypertension, myocardial infarction, and rheumatoid arthritis were identified using standard algorithms at ICES, which are based on validated algorithms using data from physician visits, emergency department visits and hospitalizations [32–37]. The five-year prevalence of each chronic disease was estimated. Health service use outcomes included primary care physician visits, specialist physician visits, emergency department visits, hospitalizations, and home care visits, and were derived from the databases described above. Primary care visits were defined as visits to doctors with specialties in family medicine or family medicine/emergency medicine. Specialist physician visits were defined as all other physician visits. All health services use outcomes were measured as dichotomous variables (yes/no) and counts (number of encounters within the follow-up period).

### Covariates

Covariates for models assessing chronic disease were held at their baseline status and included: age (continuous), sex, residential geography, socioeconomic status, and rurality of residence. Socioeconomic status was characterized by median community income quintile (1 = lowest to 5 = highest) using Canada Census data linked to postal codes. The Rurality Index of Ontario (RIO) [38] and participants’ postal codes were used to determine rurality of residence. For the RIO, municipalities are given a score ranging from 0–100 based on their total population, population density, and travel times to healthcare centres [39]. Using participants’ postal codes, we categorized RIO scores as major urban centres (0–9), non-major urban areas (10–30), rural areas (31–50), and rural-remote areas (51 +). Covariates for models assessing health services use also included the prevalence of asthma, COPD, diabetes, hypertension, myocardial infarction, and rheumatoid arthritis.

### Statistical analysis

Demographic characteristics between the Veteran and general populations were compared using standardized differences and variance ratios [40]. Prevalence estimates are presented for Veterans and the general population. The number and percentage of Veterans and the general population who used each health service and the median number of times those individuals accessed that resource with interquartile range are described overall. Crude and adjusted prevalence risk ratios with 95% CI were computed using logistic regression models for asthma, COPD, diabetes, myocardial infarction, rheumatoid arthritis. Modified Poisson regression with robust error variance regression models were used for hypertension. Crude and adjusted odds ratios and 95% confidence intervals were estimated for health service use dichotomous outcomes using logistic regression. Crude rate ratios and 95% CI were estimated for the count of each health services use outcome using Poisson models with a log link. Amount of follow-up time was included as an offset in the models. Prevalence ratios were adjusted for matching variables (baseline age, sex, residential geography, neighbourhood median income quintile) and rurality. Odds and rate ratios of health services use were further adjusted for the presence of measured chronic diseases. Stratified effect estimates were calculated for males and females. Two-sided hypothesis tests were completed, and P-values less than 0.05 were considered statistically significant. All analyses were performed using SAS 9.3 [41].

### Sensitivity analyses

We created matched comparator cohorts using hard matching with replacement on age and sex alone, as well as on age, sex, and residential geography for comparability with other studies contrasting Veteran health with the general population [42]. We also restricted the cohort to those who had at least one year of follow up. In both of these analyses, we repeated the analytic plan described above.

## Results

**Fig. 1:**
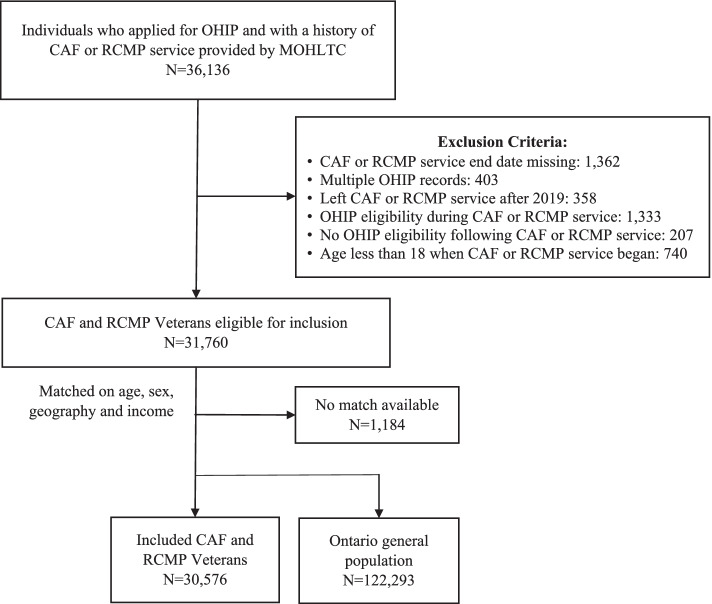
Flow chart for Veteran cohort creation OHIP: Ontario Health Insurance Plan; MOHLTC: Ministry of Health and Long-Term Care; CAF: Canadian Armed Forces; RCMP: Royal Canadian Mounted Police

A total of 36,163 Veterans were eligible for inclusion in this study and, after applying the exclusion criteria, the study group comprised 31,760 individuals (Fig. 1). Of these, 30,576 Veterans were age-, sex-, geography- and income-matched to 122,293 residents of the general population who did not have a record of a long-term care stay, had not been admitted to a rehabilitation facility, and had not received disability or income support during the period in which they would have been eligible for military service (matching rate 96.8%). Among Veterans, 14.7% were female and more than half left the CAF or RCMP at the age of 40 or older. In terms of distribution across the study period, 17.3% of the study group left the CAF or RCMP between 1990–1995, 18.5% between 1996–2000, 14.8% between 2001–2005, 17.1% between 2006–2010, 16.5% between 2011 and 2015 and 15.9% between 2016 and 2019. Overall, 51% of Veterans served for twenty or more years, 16.8% for 10–19 years, 13.8% for five to nine years, and 18.4% served less than five years. Table 1 presents the baseline demographic characteristics of Canadian Veterans living in Ontario and their age-, sex-, geography- and income-matched comparisons from the general population. Overall, 0.5% of both the Veteran and the general population groups died during the study timeframe. Median follow-up time was five years in both groups.

**Table 1:** Sociodemographic characteristics of Veterans and age, sex, region of residence and income matched general population comparison cohort (*n* = 152,869)

**Demographic characteristics**	**Veterans** (*n* = 30,576)	**General population** (*n* = 122,293)	**SD**	**VR**
**Average age in years (SD)**	41.9 (10.3)	41.9 (10.3)	0	1
**Age categories (years)**				
< 30	4,882 (16.0%)	19,475 (15.9%)	0	4.01
30–39	6,765 (22.1%)	27,098 (22.2%)	0	4
40–49	10,299 (33.7%)	41,322 (33.8%)	0	3.99
50 +	8,630 (28.2%)	34,398 (28.1%)	0	4.01
**Female sex**	4,509 (14.7%)	18,031 (14.7%)	0	4
**Region of residence**				
Erie St. Clair	497 (1.6%)	1,988 (1.6%)	0	4
South West	1,199 (3.9%)	4,796 (3.9%)	0	4
Waterloo Wellington	462 (1.5%)	1,848 (1.5%)	0	4
Hamilton Niagara Haldimand Brant	904 (3.0%)	3,616 (3.0%)	0	4
Central West	227 (0.7%)	908 (0.7%)	0	4
Mississauga Halton	387 (1.3%)	1,548 (1.3%)	0	4
Toronto Central	295 (1.0%)	1,180 (1.0%)	0	4
Central	621 (2.0%)	2,483 (2.0%)	0	4
Central East	746 (2.4%)	2,984 (2.4%)	0	4
South East	6,382 (20.9%)	25,523 (20.9%)	0	4
Champlain	15,215 (49.8%)	60,856 (49.8%)	0	4
North Simcoe Muskoka	2,374 (7.8%)	9,495 (7.8%)	0	4
North East	1,115 (3.6%)	4,460 (3.6%)	0	4
North West	152 (0.5%)	608 (0.5%)	0	4
**Median community income quintile**				
1 (lowest)	3,358 (11.0%)	13,430 (11.0%)	0	4
2	5,381 (17.6%)	21,522 (17.6%)	0	4
3	6,642 (21.7%)	26,567 (21.7%)	0	4
4	7,914 (25.9%)	31,655 (25.9%)	0	4
5 (highest)	7,281 (23.8%)	29,119 (23.8%)	0	4
Rurality of residence*				
0–9	17,676 (57.8%)	73,152 (59.8%)	0.04	4.06
10–30	5,691 (18.6%)	17,275 (14.1%)	0.12	4.99
31–50	3,972 (13.0%)	24,786 (20.3%)	0.2	2.8
51 +	3,237 (10.6%)	7,080 (5.8%)	0.18	6.94

*SD* Standardized differencesn, *VR* Variance ratio

^*^increasing values indicate increasing rurality as measured by a combination of population size and access to health resources

Table 2 describes the prevalence of asthma, COPD, hypertension, diabetes, myocardial infarction, and rheumatoid arthritis in Veterans in the five years following release and in the primary matched general population and summarizes the prevalence risk ratios for each chronic disease in Veterans compared to the general population. After adjusting for confounders, Veterans had a significantly lower prevalence of all measured chronic diseases than the general population, ranging from a 68% lower prevalence of COPD to a 24% lower prevalence of myocardial infarction during the first five years following release.

**Table 2:** Unadjusted and adjusted risk ratios of chronic disease (Reference: age-, sex-, geography- and median community income-matched general population cohort)

	Number of events (%)		Crude risk ratio (95% CI)	*p*	Adjusted risk ratio* (95% CI)	*p*
	**Veterans** (*n* = 30,576)	**General Population** (*n *= 122,293)				
**Asthma**	1,561 (5.1%)	11,763 (9.6%)	0.51 (0.48–0.53)	< .0001	0.50 (0.48–0.53)	< .0001
**COPD**	470 (1.5%)	5,631 (4.6%)	0.32 (0.29–0.36)	< .0001	0.32 (0.29–0.36)	< .0001
**Hypertension**	3,768 (12.3%)	21,163 (17.3%)	0.71 (0.69–0.74)	< .0001	0.74 (0.71–0.76)	< .0001
**Diabetes**	1,411 (4.6%)	7,688 (6.3%)	0.72 (0.68–0.76)	< .0001	0.71 (0.67–0.76)	< .0001
**Myocardial infarction**	134 (0.4%)	705 (0.6%)	0.76 (0.63–0.91)	0.004	0.76 (0.63–0.92)	0.004
**Rheumatoid arthritis**	104 (0.3%)	550 (0.4%)	0.76 (0.61–0.93)	0.009	0.74 (0.60–0.92)	0.006

*CI* Confidence intervals, *COPD* Chronic obstructive pulmonary disease

^*^adjusted for age, sex, region of residence, median community income quintile and rurality through matching and inclusion of covariates in the statistical model

Table 3 describes the proportion of Veterans and matched cohort who had at least one physician visit, emergency department visit, hospitalization, or homecare visit in the five years following release and summarizes the relative risk ratios comparing Veterans to the general population. Odds ratios increased in magnitude when comorbidities were added to the models as a means of adjusting for health service need. After adjusting for confounders, the odds of a primary care visit were 76% higher for Veterans compared to the general population and 39% higher for a specialist physician visit. Veterans had 5% lower odds of having at least one visit to the emergency department and were as likely as the general population to have a hospital admission or receive a homecare visit.

**Table 3:** Odds ratios of health care visits, by visit type and Veteran status

Visit type	N (%)	Crude odds ratio (95% CI)	*p*	Adjusted odds ratio* (95% CI)	*p*	Adjusted odds ratio ** (95% CI)	*p*
**Primary care physician visits**							
Veterans	26,268 (85.9)	1.61 (1.55–1.66)	< 0.0001	1.63 (1.57–1.69)	< 0.001	1.76 (1.70–1.83)	< 0.001
Gen Pop	96,806 (79.2)	1.00 (ref)		1.00 (ref)		1.00 (ref)	
**Specialist physician visits**							
Veterans	18,507 (60.5)	1.27 (1.24–1.30)	< 0.0001	1.28 (1.25–1.31)	< 0.0001	1.39 (1.35–1.42)	< 0.001
Gen Pop	66,973 (54.8)	1.00 (ref)		1.00 (ref)		1.00 (ref)	
**Emergency department visits**							
Veterans	12,875 (42.1)	0.91 (0.88–0.93)	< 0.0001	0.89 (0.86–0.91)	< 0.0001	0.95 (0.92–0.97)	< 0.0001
Gen Pop	54,453 (44.5)	1.00 (ref)		1.00 (ref)		1.00 (ref)	
**Hospitalizations**							
Veterans	3,236 (10.6)	0.90 (0.86–0.93)	< 0.0001	0.88 (0.85–0.92)	< 0.0001	0.99 (0.95–1.03)	0.50
Gen Pop	14,272 (11.7)	1.00 (ref)		1.00 (ref)		1.00 (ref)	
**Home care visits**							
Veterans	900 (2.9)	0.86 (0.80–0.93)	< 0.0001	0.86 (0.80–0.92)	< 0.0001	0.97 (0.90–1.04)	0.39
Gen Pop	4,147 (3.4)	1.00 (ref)		1.00 (ref)		1.00 (ref)	

*N* Number of events, *CI* Confidence interval, ref Reference group, gen pop General population

^*^Adjusted for age, sex, region of residence, income, rurality

^**^Adjusted for age, sex, region of residence, income, rurality, asthma, COPD, hypertension, diabetes mellitus, myocardial infarction and rheumatoid arthritis

Table 4 describes the median number of each healthcare encounter among those with at least one visit in the five years following release and summarizes the relative rate ratios comparing rates of health services use between Veterans and the general population. After adjusting for confounders, Veterans had a slightly higher relative rate of primary care physician visits, specialist physician visits, and emergency department visits than the general population, ranging from 6–9% higher. Hospitalization and home care rates were similar between groups.

**Table 4:** Relative rate ratios of health care visits, by visit type and Veteran status

**Visit type**	Median # visits (IQR)	Crude rate ratio (95% CI)	*p*	Adjusted rate ratio* (95% CI)	*p*	Adjusted rate ratio** (95% CI)	*p*
**Primary care physician visits**							
Veterans	9 (4–16)	1.02 (1.00–1.03)	0.05	1.01 (1.00–1.03)	0.09	1.09 (1.07–1.10)	< 0.0001
Gen Pop	9 (4–16)	1.00 (ref)		1.00 (ref)		1.00 (ref)	
**Specialist physician visits**							
Veterans	4 (2–9)	1.00 (0.97–1.03)	0.92	1.00 (0.98–1.03)	0.97	1.06 (1.03–1.08)	< 0.0001
Gen Pop	4 (2–9)	1.00 (ref)		1.00 (ref)		1.00 (ref)	
**Emergency department visits**							
Veterans	2 (1–3)	1.04 (1.02–1.07)	0.0006	1.02 (0.99–1.05)	0.13	1.06 (1.03–1.08)	< 0.0001
Gen Pop	2 (1–3)	1.00 (ref)		1.00 (ref)		1.00 (ref)	
**Hospitalizations**							
Veterans	1 (1–2)	1.00 (0.97–1.04)	0.92	1.00 (0.97–1.04)	0.92	1.03 (1.00–1.08)	0.06
Gen Pop	1 (1–2)	1.00 (ref)		1.00 (ref)		1.00 (ref)	
**Home care visits**							
Veterans	9 (4–26)	0.90 (0.67–1.20)	0.47	0.89 (0.67–1.18)	0.42	0.93 (0.69–1.26)	0.65
Gen Pop	11 (5–30)	1.00 (ref)		1.00 (ref)		1.00 (ref)	

IQR: interquartile range; CI: confidence interval; ref: reference group; gen pop: general population

^*^Adjusted for age, sex, region of residence, income, rurality

^**^Adjusted for age, sex, region of residence, income, rurality, asthma, COPD, hypertension, diabetes mellitus, myocardial infarction and rheumatoid arthritis

### Stratification by sex

Comparisons between Veteran and general population health and health services use were stratified by sex (Supplementary Tables 1–3). For both sexes, effect estimates for Veterans compared to general population aligned with the main effects presented in Tables 2, 3 and 4 albeit more closely among males than females.

### Sensitivity analyses

Results were robust to comparisons with an age- and sex-matched comparison group and with an age-, sex- and geography-matched comparator group (results available from authors). Results were also robust to excluding those with less than one year of potential follow-up data (results available from authors).

## Discussion

Military service places greater physical and mental stress on its members than many other occupations and there is potential for members of the service to be exposed to environmental stimuli and/or physical conditions that could exacerbate chronic illness. On the other hand, a healthy worker effect, i.e., a tendency among people who are actively employed to be healthier [43], would not be unexpected in military Veterans, particularly in the first five-years following release. Our study documented a lower prevalence of asthma, COPD, hypertension, diabetes, myocardial infarction, and rheumatoid arthritis in Canadian Veterans relative to the general population. However, these findings differ from much of the published literature in this field of research. For example, in 2019, the Life After Service Studies (LASS) research program, which collects survey data from Canadian Veterans on their transition to civilian life, reported a higher lifetime prevalence of asthma among Veterans compared to civilians, and noted that self-reported rates of lifetime high blood pressure, depression, and anxiety were increasing over time [44]. Among Veterans in Scotland, the prevalence of cardiovascular disease (acute myocardial infarction, peripheral arterial disease, stroke) after a mean follow-up period of 38 years is slightly higher [21] and the prevalence of COPD after a mean follow-up period of 28 years is similar between Veterans and civilians [45]. In the US, Veterans have been reported to have higher age and sex standardized lifetime prevalence of diabetes [46, 47], rheumatoid arthritis and hypertension (the latter two linked to PTSD) [48, 49] compared to civilians. Reasons for the differences between our findings and other reports could include the methods by which the data were collected (e.g., in studies using survey data, people are more likely to respond if they have a complaint or concern, or if they require more services), analyzed (differences in results with direct comparisons versus the use of standardization), or variation in the specific study cohort or timeframe examined. Our study limited the time period of interest to the five years after release, whereas many other studies cited here report on Veterans’ long-term health outcomes. Future research could focus on routinely collected administrative health data so that we can better describe Veterans’ need for support related to chronic illnesses, and on comparing the quality and quantity of healthcare Veterans with a diagnosis receive in comparison to the general population. These studies would provide a more robust and comprehensive understanding of potential gaps in care.

Despite lower prevalence of common chronic illnesses, our study found the odds of using health services were higher or similar for Veterans compared to the general population. In particular, we documented higher rates of primary care and specialist physician visits and lower rates of emergency department visits among Veterans. Hospitalization rates and rates of receiving home care services were similar in both groups. Veterans’ health service use patterns reported in the literature are mixed. In the US, higher health service use among Veterans is often linked to elevated likelihood of adverse health outcomes, particularly among older Veterans: risk factors like smoking and alcohol misuse and higher rates of cardiovascular disease [50], mental disorders [50], and osteoarthritis [51] have been identified as drivers of this higher service use, as have higher frailty scores [52]. Veterans in New Zealand are reported to have higher hospitalization rates than the general public [53]; in the UK, Veterans with a self-reported mental health problem are more likely to be admitted to hospital for a chronic condition (e.g., hypertension, gastrointestinal disorders, joint disorders) [54]. There is also substantial concern about lack of access to mental health services and stigma in seeking mental health care [55]. Our study results, then, present something of a paradox in that lower rates of illness occurred in a population with higher health service use. This finding may reflect visits to physicians for conditions we didn’t measure in this study but are commonly associated with occupational military stressors, such as musculoskeletal injuries, hearing loss, or other operational injury such as traumatic brain injury [56–58]. Increased likelihood of visiting a physician among Veterans may relate to military-specific help-seeking behaviours resulting from a culture of maintaining peak condition of health to ensure operational readiness. Our findings may also represent an example of achieving good health through increased use of health services, surveillance, and monitoring, supported by the observed lower likelihood of visiting the emergency department but contradicted by similar rates of hospitalization. Further research exploring these potential explanations is warranted to understand why differences exist between Veterans and the general population and to determine whether the observed healthier status of Veterans can be sustained over time and/or be translated to the general population.

This study contributes novel information on Canadian Veterans’ health and health services use with important implications for health system and public health planners. Like civilians, Veterans are users of the Canadian healthcare system and must be accounted for when considering the overall health of the population and how health system resources are distributed. Veterans may leave military service with better health than the general population, but there is little understanding of military culture among healthcare providers [59, 60], making it difficult for the existing health system to support Veterans in maintaining this apparent advantage. Further studies are needed to examine in more detail what specific factors contribute to maintaining the health of Veterans and to identify conditions and illnesses more prevalent in this group, as well as opportunities to intervene on these conditions. Lessons learned from the health and health care use patterns of military Veterans could then be applied in the public health system to improve outcomes in the general population of Canada and support overall population health. As well, further analysis of age, sex and gender differences in Veteran outcomes will be important as our understanding of required supports for members of the military and their families continues to grow [61, 62].

### Study limitations

Although the use of population-based administrative health data has many advantages, there are also some limitations related to the datasets used in this study that are worth noting. Veterans Affairs Canada estimates that approximately 1,050 Veterans are released from the CAF and take up civilian residence in Ontario each year [25]. While the true denominator of Veterans living in Ontario during our study timeframe is not known, the number of Veterans entering our cohort per year approximated the expected number of Veterans who take up residence in Ontario annually. Further, the overall age, sex, and length of service distribution in our cohort is similar to that reported by Veterans Affairs Canada with a slight exception: our cohort has fewer younger veterans (< 25 years old) and a larger number of older veterans (50 and older) than expected. This is likely explained by the inclusion in our cohort of RCMP Veterans who released between January 1, 1990 and March 31, 2013 [30]. We are unable to study CAF and RCMP Veterans who released during this timeframe separately as the MOHLTC includes both under a single Veteran status identifier variable. However, the majority of our cohort are likely to be Veterans of the CAF rather than the RCMP, as fewer than 200 RCMP Veterans take up residence in Ontario per year [30] and after March 31, 2013, only CAF Veterans were included in the cohort. Finally, we were only able to examine six chronic conditions in this study, but Veterans may experience other illnesses at higher rates than members of the Ontario general population. Further research is needed to validate additional chronic disease algorithms to support future investigations into other conditions. Routinely collected health administrative data do not include information on lifestyle factors that may explain differences in chronic disease prevalence, such as smoking, alcohol use, or physical activity levels. Future research comparing these lifestyle factors between Veterans and non-Veterans over time would provide context to these relative measures of disease.

## Conclusions

Our study found that more Veterans visited a physician visit than the general population, a striking finding given the lower burden of comorbidities amongst Veterans. While this may reflect a need for services for diagnoses, disabilities or illnesses we did not measure in our study, our findings suggest that Canadian Veterans have good access to primary and specialty health care. However, there continues to be room for improvement and lessons that could be learned from Veteran health and health service use patterns. Calls to action to better support CAF Veterans have included increasing cultural competency among healthcare providers [59], increasing awareness of specialized healthcare services funded by VAC that eligible Veterans could be referred for [63], and enhancing collaboration and communication between healthcare providers and VAC staff to ensure continuity of care [64]. In order to ensure Canadian Veterans are truly well supported health system planners, analysts and researchers should take careful account of this population for both health system resources and health services research. The reasons for our findings in light of contradictory findings in other jurisdictions also warrant further study, and lessons learned could be applied in the public health system to improve outcomes in the general population of Canada.

## Supplementary Information

**Additional file 1: Supplementary Table 1.** Unadjusted and adjusted risk ratios of chronic disease stratified by sex (reference: age-, sex-, geography- and median community income-matched general population cohort).** Supplementary Table 2.** Relative odds ratios of health care visits, by sex, visit type and Veteran status.** Supplementary Table 3.** Relative rate ratios of health care visits, by sex, visit type and Veteran status.

## Data Availability

The data set from this study is held securely in coded form at ICES. While data sharing agreements prohibit ICES from making the data set publicly available, access may be granted to those who meet pre-specified criteria for confidential access, available at www.ices.on.ca/DAS. The full data set creation plan and underlying analytic code are available from the authors upon request, understanding that the programs may rely upon coding templates or macros that are unique to ICES.

## Abbreviations

CAF: Canadian armed forces
CI: Confidence interval
CIHI: Canadian institute of health information
COPD: Chronic obstructive pulmonary disease
MOHLTC: Ministry of health and long-term care
OHIP: Ontario health insurance plan
RCMP: Royal Canadian mounted police
RIO: Rurality index of Ontario
UK: United Kingdom
US: United States
VAC: Veterans affairs Canada
