# Supplementary material for: Canadian Veteran chronic disease prevalence and health services use in the five years following release: a matched retrospective cohort study using routinely collected data
Source: BMC Public Health. 2022 Sep 5;22:1678. doi: 10.1186/s12889-022-14053-4 (PMC9442935; doi:10.1186/s12889-022-14053-4)
Supplement: Supplementary file 1 — Additional file 1: Supplementary Table 1. Unadjusted and adjusted risk ratios of chronic disease stratified by sex (reference: age-, sex-, geography- and median community income-matched general population cohort). Supplementary Table 2. Relative odds ratios of health care visits, by sex, visit type and Veteran status. Supplementary Table 3. Relative rate ratios of health care visits, by sex, visit type and Veteran status. [file 12889_2022_14053_MOESM1_ESM.docx]

| Supplementary Table 1. Unadjusted and adjusted risk ratios of chronic disease – stratified by sex  (reference: age-, sex-, geography- and median community income-matched general population cohort) | | | | | | |
| --- | --- | --- | --- | --- | --- | --- |
|  | **Number of events (%)** | | **Crude risk ratio**  **(95% CI)** | **p-value** | **Adjusted risk ratio**  **(95% CI)** | **p-value** |
|  | **Male Civilians** | **Male Veterans** |  |  |  |  |
| Asthma | 9441 (9.1) | 1260 (4.8) | 0.51 (0.48-0.54) | <0.0001 | 0.51 (0.48-0.54) | <0.0001 |
| COPD | 4953 (4.8) | 409 (1.6) | 0.32 (0.29-0.35) | <0.0001 | 0.32 (0.29-0.36) | <0.0001 |
| Diabetes | 6999 (6.7) | 1295 (5.0) | 0.73 (0.68-0.77) | <0.0001 | 0.72 (0.68-0.77) | <0.0001 |
| Myocardial infarction | 692 (0.7) | 132 (0.5) | 0.76 (0.63-0.92) | 0.004 | 0.77 (0.64-0.92) | 0.005 |
| Rheumatoid arthritis | 404 (0.4) | 77 (0.3) | 0.76 (0.60-0.97) | 0.03 | 0.75 (0.58-0.95) | 0.019 |
|  | **Number of events (%)** | | **Crude risk ratio**  **(95% CI)** | **p-value** | **Adjusted risk ratio**  **(95% CI)** | **p-value** |
|  | **Female Civilians** | **Female Veterans** |  |  |  |  |
| Asthma | 2322 (12.9) | 301 (6.7) | 0.48 (0.43-0.55) | <0.0001 | 0.50 (0.43-0.56) | <0.0001 |
| COPD | 678 (3.8) | 61 (1.4) | 0.35 (0.27-0.46) | <0.0001 | 0.35 (0.27-0.45) | <0.0001 |
| Diabetes | 689 (3.8) | 116 (2.6) | 0.67 (0.54-0.81) | <0.0001 | 0.66 (0.54-0.81) | <0.0001 |
| Myocardial infarction | 13 (0.1) | ≤ 5 | 0.62 (0.14-2.73) | 0.52 | 0.58 (0.11-2.98) | 0.51 |
| Rheumatoid arthritis | 146 (0.8) | 27 (0.6) | 0.74 (0.49-1.11) | 0.15 | 0.73 (0.49-1.10) | 0.14 |
| CAF: Canadian Armed Forces; CI: confidence intervals; COPD: chronic obstructive pulmonary disease; RCMP: Royal Canadian Mounted Police  *adjusted for age, region of residence, median community income quintile and rurality through matching and inclusion of covariates in the statistical model | | | | | | |

| Supplementary Table 2: Relative odds ratios of health care visits, by sex, visit type and Veteran status | | | | | | | | |
| --- | --- | --- | --- | --- | --- | --- | --- | --- |
| Visit type | **Number of events (%)** | | **Crude odds ratio**  **(95% CI)** | **p-value** | **Adjusted odds ratio***  **(95% CI)** | **p-value** | **Adjusted odds ratio****  **(95% CI)** | **p-value** |
|  | **Male Veterans** | **Male Civilians** |  |  |  |  |  |  |
| Primary care physician visits | 22294 (85.5) | 81230 (77.9) | 1.68 (1.61-1.74) | <0.0001 | 1.70 (1.63-1.76) | <0.0001 | 1.83 (1.76-1.90) | <0.0001 |
| Specialist physician visits | 15466 (59.3) | 55028 (52.8) | 1.31 (1.27-1.34) | <0.0001 | 1.32 (1.28-1.36) | <0.0001 | 1.43 (1.39-1.47) | <0.0001 |
| ED visits | 10925 (41.9) | 46366 (44.5) | 0.90 (0.88-0.93) | <0.0001 | 0.89 (0.86-0.91) | <0.0001 | 0.94 (0.92-0.97) | <0.0001 |
| Hospitalizations | 2407 (9.2) | 10804 (10.4) | 0.88 (0.84-0.92) | <0.0001 | 0.88 (0.83-0.92) | <0.0001 | 0.99 (0.94-1.04) | 0.65 |
| Home care visits | 739 (2.8) | 3548 (3.4) | 0.83 (0.76-0.90) | <0.0001 | 0.83 (0.76-0.90) | <0.0001 | 0.94 (0.86-1.02) | 0.11 |
| Visit type | **Number of events (%)** | | **Crude odds ratio**  **(95% CI)** | **p-value** | **Adjusted odds ratio***  **(95% CI)** | **p-value** | **Adjusted odds ratio****  **(95% CI)** | **p-value** |
|  | **Female Veterans** | **Female Civilians** |  |  |  |  |  |  |
| Primary care physician visits | 3974 (88.1) | 15576 (86.4) | 1.17 (1.06-1.29) | 0.002 | 1.19 (1.08-1.32) | 0.0006 | 1.32 (1.19-1.46) | <0.0001 |
| Specialist physician visits | 3041 (67.4) | 11945 (66.2) | 1.06 (0.99-1.13) | 0.13 | 1.06 (0.99-1.14) | 0.09 | 1.16 (1.08-1.24) | <0.0001 |
| ED visits | 1950 (43.2) | 8087 (44.8) | 0.94 (0.88-1.00) | 0.05 | 0.88 (0.82-0.94) | 0.0003 | 0.95 (0.89-1.02) | 0.19 |
| Hospitalizations | 829 (18.4) | 3468 (19.2) | 0.95 (0.87-1.03) | 0.20 | 0.92 (0.84-1.01) | 0.07 | 0.98 (0.89-1.07) | 0.61 |
| Home care visits | 161 (3.6) | 599 (3.3) | 1.08 (0.90-1.29) | 0.41 | 1.05 (0.88-1.25) | 0.61 | 1.16 (0.97-1.39) | 0.11 |
| *Adjusted for age, region of residence, median community income, rurality  **Adjusted for age, region of residence, income, rurality, asthma, COPD, hypertension, diabetes mellitus, myocardial infarction and rheumatoid arthritis | | | | | | | | |

| Supplementary Table 3: Relative rate ratios of health care visits, by sex, visit type and Veteran status | | | | | | | | |
| --- | --- | --- | --- | --- | --- | --- | --- | --- |
| Visit type | **Median #** **visits (IQR)** | | **Crude rate ratio**  **(95% CI)** | **p-value** | **Adjusted rate ratio***  **(95% CI)** | **p-value** | **Adjusted rate ratio****  **(95% CI)** | **p-value** |
|  | **Male Veterans** | **Male**  **Civilians** |  |  |  |  |  |  |
| Primary care physician visits | 8.0 (4.0-15.0) | 8.0 (4.0-15.0) | 1.02 (1.00- 1.03) | 0.03 | 1.01 (0.99- 1.02) | 0.37 | 1.08 (1.07- 1.10) | <0.0001 |
| Specialist physician visits | 4.0 (2.0- 9.0) | 4.0 (2.0- 9.0) | 0.98 (0.95- 1.01) | 0.14 | 0.97 (0.95- 1.00) | 0.05 | 1.03 (1.00- 1.06) | 0.04 |
| ED visits | 2.0 (1.0- 3.0) | 2.0 (1.0- 3.0) | 1.04 (1.01- 1.06) | 0.01 | 1.02 (0.99- 1.04) | 0.22 | 1.05 (1.02- 1.08) | 0.0002 |
| Hospitalizations | 1.0 (1.0- 2.0) | 1.0 (1.0- 2.0) | 0.99 (0.95- 1.03) | 0.59 | 0.99 (0.95- 1.04) | 0.70 | 1.03 (0.99- 1.08) | 0.16 |
| Home care visits | 9.0 (5.0-26.0) | 11.0 (5.0-30.0) | 0.92 (0.67- 1.28) | 0.64 | 0.92 (0.66- 1.26) | 0.59 | 0.97 (0.69- 1.36) | 0.84 |
| Visit type | **Median # visits (IQR)** | | **Crude rate ratio**  **(95% CI)** | **p-value** | **Adjusted rate ratio***  **(95% CI)** | **p-value** | **Adjusted rate ratio****  **(95% CI)** | **p-value** |
|  | **Female Veterans** | **Female**  **Civilians** |  |  |  |  |  |  |
| Primary care physician visits | 12.0 (6.0-21.0) | 12.0 (6.0-22.0) | 1.03 (1.00- 1.07) | 0.08 | 1.04 (1.00- 1.07) | 0.04 | 1.10 (1.06- 1.13) | <0.0001 |
| Specialist physician visits | 6.0 (2.0-12.0) | 5.0 (2.0-12.0) | 1.11 (1.04- 1.18) | 0.002 | 1.12 (1.05- 1.20) | 0.0006 | 1.17 (1.09- 1.25) | <0.0001 |
| ED visits | 2.0 (1.0- 4.0) | 2.0 (1.0- 3.0) | 1.09 (1.02- 1.17) | 0.01 | 1.03 (0.96- 1.11) | 0.40 | 1.08 (1.00- 1.16) | 0.05 |
| Hospitalizations | 1.0 (1.0- 2.0) | 1.0 (1.0- 2.0) | 1.05 (0.99- 1.10) | 0.10 | 1.04 (0.98- 1.10) | 0.17 | 1.05 (0.99- 1.11) | 0.08 |
| Home care visits | 8.0 (4.0-21.0) | 10.0 (4.0-27.0) | 0.78 (0.43- 1.42) | 0.42 | 0.76 (0.41- 1.39) | 0.37 | 0.79 (0.43- 1.46) | 0.46 |

*Adjusted for age, region of residence, median community income, rurality

**Adjusted for age, region of residence, income, rurality, asthma, COPD, hypertension, diabetes mellitus, myocardial infarction and rheumatoid arthritis
